# Interplacental uterine expression of genes involved in prostaglandin synthesis during canine pregnancy and at induced prepartum luteolysis/abortion

**DOI:** 10.1186/1477-7827-12-46

**Published:** 2014-05-30

**Authors:** Mariusz P Kowalewski, Ewa Kautz, Elisabeth Högger, Bernd Hoffmann, Alois Boos

**Affiliations:** 1 Institute of Veterinary Anatomy, Vetsuisse Faculty, University of Zurich, Zurich, Switzerland; 2 Clinic for Obstetrics, Gynecology and Andrology of Large and Small Animals, Justus-Liebig University Giessen, Giessen, Germany

**Keywords:** Canine uterus, Prostaglandins

## Abstract

**Background:**

In the non-pregnant dog, ovarian cyclicity is independent of a uterine luteolysin. This is in contrast to pregnant animals where a prepartum increase of luteolytic PGF2α occurs, apparently originating in the pregnant uterus. Recently, the placenta as a source of prepartum prostaglandins (PGs) was investigated, indicating fetal trophoblast cells as the likely main source. However, the possible contribution of uterine interplacental tissues to the production of these hormones has not yet been thoroughly examined in the dog.

**Methods:**

Several key factors involved in the production and/or actions of PGs were studied: cyclooxygenase 2 (COX2, PTGS2), PGF2α-synthase (PGFS/AKR1C3), PGE2-synthase (PGES), and the respective receptors FP (PTGFR), EP2 (PTGER2) and EP4 (PGTER4), 15-hydroxyprostaglandin dehydrogenase (HPGD), PG-transporter (PGT, SLCO2A1) and progesterone receptor. Their expression and localization patterns were assessed by Real Time PCR and immunohistology in the interplacental uterine sites from pregnant dogs during the pre-implantation period (days 8–12), post-implantation (days 18–25), mid-gestation (days 35–40) and during antigestagen-induced luteolysis/abortion.

**Results:**

Whereas only low COX2 expression was observed in uterine samples at all the selected time points, expression of PGFS/AKR1C3 strongly increased post-implantation. A gradual increase in *PGES*-mRNA expression was noted towards mid-gestation. *FP*-mRNA expression decreased significantly with the progression of pregnancy until mid-gestation. This was associated with clearly detectable expression of *HPGD*, which did not change significantly over time. The expression of *FP* and *EP2*-mRNA decreased significantly over time while *EP4-*mRNA expression remained unaffected. The antigestagen-treatment led to a significant increase in expression of *COX2*, *PGES*, *EP2* and *PGT* (*SLCO2A1*) mRNA. COX2 was localized predominantly in the myometrium. The expression of PGFS/AKR1C3, which was unchanged, was localized mostly to the surface luminal epithelium. The expression of *EP4*, *PGT* and *HPGH* did not change during treatment, they were co-localized with PGES and EP2 in all uterine compartments.

**Conclusions:**

The data clearly demonstrate the basic capability of the canine pregnant uterus to produce and respond to PGs and suggests their functions both as local regulatory factors involved in the establishment and maintenance of pregnancy, as well as potential contributors to the process of parturition, supporting the myometrial contractility associated with fetal expulsion.

## Background

In the domestic dog (Canis familiaris), the establishment and maintenance of pregnancy depend on the luteal provision of progesterone (P4) as the only source of this hormone both during gestation and in the non-pregnant cycle [1,2]. Luteal function is similar in both pregnant and non-pregnant bitches (see reviews in [3-5]). The circulating P4 levels start to deviate strongly only shortly prior to term on days 62/63, when a steep decline marks onset of prepartum luteolysis 12-24 h before the first clinical signs of labour become visible [6,7]. In non-pregnant animals, in contrast, luteal regression continues beyond this point of time and circulating P4 concentrations decrease slowly until levels below 1 ng/ml are reached about 10 to 20 days later and the cycle enters the phase of obligatory sexual quiescence, i.e., anoestrus [6]. Consequently, in contrast to what is observed in non-pregnant dogs, prepartum luteolysis appears to be a process governed by active regulatory mechanisms. In respect to luteolytic factors, normal cyclic ovarian steroidogenic function has been observed in hysterectomized bitches, as in cats and primates, rendering luteal function as independent of a uterine luteolysin (prostaglandin (PG) F2α; PGF2α) in these species [8,9]. This is in contrast to livestock, where ovarian cyclicity is maintained due to periodic production and secretion of uterine PGF2α.

In dogs, prostaglandins synthesized locally within the corpus luteum appear to be involved in luteal maintenance rather than in luteolysis/regression, as indicated by increased expression of cyclooxygenase 2 (COX2, PTGS2) and PGE2-synthase (PGES) at the beginning of the canine luteal phase [10-12]. This hypothesis has been substantiated recently by showing a luteotrophic role of PGE2 in canine lutein cells *in vitro*. Acting mostly at the level of substrate provision facilitated by steroidogenic acute regulatory (STAR) protein expression and function, and not at the level of activity of the two major steroidogenic enzymes, 3βHSD and P450scc enzyme, PGE2 significantly increased progesterone output in lutein cells [13].

In pregnant animals the prepartum luteolysis is concomitant with a rapid increase of PGF2α in maternal peripheral blood, implying its involvement during prepartum luteolysis and/or fetal expulsion [14-16]. With regard to the endocrine mechanisms regulating the process of parturition in the dog, most of the currently available knowledge relates to the endocrine capabilities of the utero-placental compartments, comprising the placenta with its adjacent uterine tissues [15,17-20]. Recent investigations into the expression of factors involved in prostaglandin synthesis and function, the so-called PG-system, have indicated an upregulated expression of COX2 in fetal trophoblast cells as the possible origin of prepartum PGF2α release within the utero-placental compartment [15]. This finding corroborates the observation from the same study that interfering with P4 receptor (PGR) function by application of an antigestagen in mid-pregnant dogs leads to upregulation of utero-placental COX2 expression, mostly in the fetal trophoblast, and results in significantly elevated peripheral PGF2α concentrations. The only known canine-specific PGF2α-synthase (PGFS), classified as aldo-keto reductase family 1, member C3 (AKR1C3) which is responsible for the direct conversion of PGH2 to PGF2α, was localized in epithelial cells of the superficial uterine glands, the so-called glandular chambers, and in fetal trophoblasts [17]. Interestingly, however, expression of this enzyme was decreased during prepartum luteolysis. This was paralleled by the reduced expression of 15-hydroxyprostaglandin dehydrogenase (HPGD), an enzyme catabolising PGF2α and PGE2 to their inactive metabolites [17]. Together with the concomitantly increased expression of *PGE2*-synthase (PGES) [15], a possible functional interrelationship between these entities has been suggested. The potential involvement of alternative pathways for PGF2α synthesis has not, however, yet been investigated. It is noteworthy that the results to date clearly show that there is no parturition-related increase in estrogens mediating the prepartum PGF2α release, and the sporadically observed elevated levels of cortisol measured in maternal blood peripartum seem not to be mandatory for normal canine parturition [1,21].

Less attention has been paid so far to uterine endocrine capabilities in the dog during pregnancy and parturition, especially those uterine sites not attached to the placenta. In one study, basal and 12,13-phorbol dibutyrate (PDBu; protein kinase C stimulator) - mediated capabilities of canine endometrial and placental explants to produce PGF2α were determined *in vitro*, revealing that the endometrium of pregnant dogs seems to acquire an increased capacity to produce PGF2α immediately prepartum, which - on a tissue weight basis of the explants used - exceeded even that of the placenta [20]. However, when taking into account the larger mass of placental tissue *in vivo*, the authors inferred that also the placenta might substantially contribute to the prepartum PGF2α release. The latter conclusion was confirmed in own studies [15] which showed an upregulation of *COX2* on the mRNA and protein level in the trophoblast prior to parturition, rather indicating that the placenta and not the endometrium is the main source of the prepartum PGF2α release. Thus, the question regarding the involvement of prostaglandins synthesized in the interplacental sites in regulating the processes of pregnancy and parturition in the bitch is still open. Such a functional interrelationship has previously been established for other species, e.g., cattle, where increased uterine, intercaruncular expression of COX2 and PGF2α-receptor (PTGFR, FP) was observed in animals with induced parturition, indicating the likely contribution of this increase to labour [22].

Therefore, with the aim of improving our understanding of uterine endocrine function during canine gestation, here, the expression and localization of factors involved in prostaglandin synthesis and its biological activity, as well as the expression of progesterone receptor (PGR), were investigated in the interplacental uterine sites. Based on the tissue material available, gestational periods from the early pre-implantation stage until fully established pregnancy (mid-gestation) were included. Additionally, in order to investigate the possible functional pathways and aiming to gain further information on the underlying endocrine mechanisms, expression of the PG-system was assessed in mid-pregnant bitches in which PGR function was blocked with an antigestagen in order to induce preterm luteolysis/abortion.

## Methods

### Animals and tissue material

As already indicated in a previous study [15], in which tissue materials from the same animals were used, all experimental procedures were carried out in accordance with animal welfare legislation ((permit no. II 25.3-19c20-15c GI 18/14 and VIG3-19c-20/15c GI 18,14 (Justus-Liebig University, Giessen) and permit no. Ankara 2006/06 (Faculty of Veterinary Medicine, University of Ankara)).

Clinically healthy, crossbreed bitches, aged 2–8 years had been assigned to the following groups during selected time points of pregnancy: pre-implantation (days 8–12, n = 5), post-implantation (days 18–25, n = 5) and mid-gestation (days 35–40, n = 5); the time of mating (day 0) was set as two days after ovulation, which was assessed by measuring peripheral P4 concentrations (5 > ng/ml) and by vaginal cytology. Following ovariohysterectomy tissue samples were collected from the utero-placental compartment (see in [15]) and the interplacental sites (full thickness of the uterine wall). The latter, together with the uterine samples collected from the pre-implantation stage of pregnancy, were used for the present study. Tissues were preserved for mRNA-analysis and immunohistochemistry as previously described [15]. The pre-implantation stage of early pregnancy was confirmed by uterine flushing of embryos. Based on the temporal developmental characteristics of canine uterine and placental tissues, the post-implantation and mid-gestation groups used in our study relate to periods of canine pregnancy characterized according to the status of utero-placental units: either their early formation (post-implantation) or their full development (mid-gestation, around day 40 of gestation) [23,24].

Abortion was induced in ten dogs (days 40–45 of pregnancy) by application of the antigestagen Aglepristone® [Alizine® Virbac, 10 mg/Kg bw (2×/24 hrs apart)]; ovariohysterectomies were performed 24 hrs (n = 5) and 72 hrs (n = 5) after the second treatment.

### Total RNA extraction, semi-quantitative RT-PCR

TRIzol® reagent (Invitrogen, Carlsbad, CA, USA) was used for total RNA isolation according to the manufacturer’s instructions and the RNA content was measured with a NanoDrop 2000C® spectrophotometer (Thermo Fisher Scientific AG, Reinach, CH). The elimination of genomic DNA contamination was achieved by DNase-treatment of all isolated RNAs with RQ1 RNase-free DNase (Promega, Dübendorf, CH) following the manufacturer’s protocol. The reverse transcriptase (RT) reaction was performed using random hexamers as primers and other reagents from Applied Biosystems (Foster City, CA, USA) as previously described [12,18]. For each RT reaction, 100 ng of DNase-treated total RNA were used. The reactions were carried out in an Eppendorf Mastercycler® (Vaudaux-Eppendrf AG, Basel, CH) under the following experimental conditions: 8 min at 21°C and 15 min at 42°C, then the reaction was stopped by incubation for 5 min at 99°C.

An automated fluorometer ABI PRISM® 7500 Sequence Detection System (Applied Biosystems) was used for semi-quantitative Real Time (TaqMan) PCR according to our previously described protocol [12,18]. All samples were run in duplicate. Autoclaved water instead of cDNA and the so-called RT-minus control (samples that were not reverse transcribed) were used for negative controls. The following 25 μl reaction mixtures were prepared containing: Fast Start Universal Probe Master Mix (ROX)® (Roche Diagnostics, Rotkreuz, CH), 200 nM TaqMan Probe, 300 nM of each primer, 12.5 μl Fast Start Universal Probe Master Mix (ROX), 5 μl cDNA corresponding to 100 nm total RNA per sample. The cycling conditions were: denaturation at 95°C for 10 min, 40 cycles at 95°C for 15 sec, and 60°C for 60 sec. The list of primers and TaqMan probes labelled with 6-carboxyfluorescein (6-FAM) and 6-carboxytetramethyl-rhodamine (TAMRA) is presented in Table 1. All primers were designed using Primer Express Software (Version 2.0, Applied Biosystems) and purchased from Microsynth (Balgach, CH). Three different reference genes were used for normalization: *GAPDH*, *18SrRNA* and cyclophilin A. The comparative CT method (ΔΔCT method) was applied for calculating relative gene expression levels according to a previously described protocol [12,18] and following the instructions of the manufacturer of the ABI PRISM® 7500 Sequence Detection System. The sample with the lowest concentration was used as a calibrator for calculating the relative expression of target genes in this procedure. The PCR reactions were set up to ensure approximately 100% efficiency of reactions. A commercially available TaqMan System (primer and probe mixture) of canine-specific *cyclophilin A* (TaqMan system) was purchased from Applied Biosystems (Prod. No. Cf03986523- gH). Selected PCR products were commercially sequenced (Microsynth) in order to confirm the specificity of amplicons.

**Table 1:** List of primers and taqman probes used for semi-quantitative RT-PCR

**Primer**	**Accession numbers**	**Primer sequence**	**Product lenght (bp)**
GAPDH (forward)	AB028142	5′-GCT GCCAAATAT GACGACATC A-3′	75bp
GAPDH (reverse)		5′-GTA GCC CAG GAT GCC TTT GAG-3′	
GAPDH (TaqMan Probe)		5′-TCC CTC CGA TGC CTG CTT CAC TAC CTT-3′	
18SrRNA (forward)	FJ797658	5′-GTC GCT CGC TCC TCT CCT ACT-3′	125bp
18SrRNA (reverse)		5′-GGC TGA CCG GGT TGG TTT-3′	
18SrRNA (TaqMan Probe)		5′-ACA TGC CGA CGG GCG CTG AC-3′	
PGR (forward)	NM_001003074	5′-CGA GTC ATT ACC TCA GAA GAT TTG TTT-3′	113bp
PGR (reverse)		5′-CTT CCA TTG CCC TTT TAA AGA AGA-3′	
PGR (TaqMan Probe)		5′-AAG CAT CAG GCT GTC ATT ATG GTG TCC TAA CTT-3′	
COX2 (forward)	HQ110882	5′-GGA GCA TAA CAG AGT GTG TGA TGT G-3′	87bp
COX2 (reverse)		5′-AAG TAT TAG CCT GCT CGT CTG GAA T-3′	
COX2 (TaqMan Probe)		5′-CGC TCA TCA TCC CAT TCT GGG TGC-3′	
PGFS/AKR1C3 (forward)	NM_001012344	5′-AGG GCT TGC CAA GTC TAT TGG-3′	74bp
PGFS/AKR1C3 (reverse)		5′-GCC TTG GCT TGC TCA GGA T-3′	
PGFS/AKR1C3 (TaqMan Probe)		5′-TCC AAC TTT AAC CGC AGG CAG CTG G-3′	
PGES (forward)	NM_001122854	5′-GTC CTG GCG CTG GTG AGT-3′	89bp
PGES (reverse)		5′-ATG ACA GCC ACC ACG TAC ATC T-3′	
PGES (TaqMan Probe)		5′-TCC CAG CCT TCC TGC TCT GCA GC-3′	
PTGFR (FP) (forward)	NM_001048097	5′-ACC AGT CGA ACA TCC TTT GCA-3′	86bp
PTGFR (FP) (reverse)		5′-GGC CAT CAC ACT GCC TAG AAA-3′	
PTGFR (FP) (TaqMan Probe)		5′-CAT GGT GTT CTC CGG TCT GTG CCC-3′	
PTGER2 (forward)	AF075602	5′- CAC CCT GCT GCT GCT TCT C-3′	78bp
PTGER2 (reverse)		5′-CGG TGC ATG CGG ATG AG-3′	
PTGER2 (TaqMan Probe)		5′-TGC TCG CCT GCA ACT TTC AGC GTC-3′	
PTGER4 (forward)	NM_001003054	5′-AAA TCA GCA AAA ACC CAG ACT TG-3′	96bp
PTGER4 (reverse)		5′-GCA CGG TCT TCC GCA GAA -3′	
PTGER4 (TaqMan Probe)		5′-ATCCGA ATT GCT GCT GTG AAC CCT ATC C-3′	
PGT (forward)	NM_001011558	5′-TGC AGC ACT AGG AAT GCT GTT C-3′	116bp
PGT (reverse)		5′-GGG CGC AGA GAA TCA TGG A-3′	
PGT (TaqMan probe)		5′-TCT GCA AAC CAT TCC CCG CGT G-3′	
HPGD (forward)	NM_001284477	5′-GGC AGC GAA TCT CAT GAA CAG-3′	93bp
HPGD (reverse)		5′-TCT TCT TTC TCA ATG GAT TCA AGGA-3′	
HPGD (TaqMan Probe)		5′-TGA ATG CCA TTT GCC CAG GCT TTG-3′	

### Data evaluation

A parametric one-way analysis of variance (ANOVA) was applied to test for an effect of the observational group on *COX2* (*PTGS2*), *PGT* (*SLCO2A1*) and *PGR* mRNA expression levels. In the case of P < 0.05, post-tests (Tukey-Kramer multiple comparison test) were performed. Due to the uneven distribution of the data obtained for uterine mRNA expression of *PGFS/AKR1C3*, *PGES*, *FP* (*PTGFR*), *EP2* and *EP4* (*PTGER2* and *PTGER4*) and *HPGD*, the Kruskal-Wallis test (a nonparametric ANOVA) followed by Dunn’s multiple comparison test were performed in the event of P < 0.05. In experiments assessing the expression of target genes after Aglepristone®-induced preterm luteolysis (abortion), Dunnett’s multiple comparison test was performed. In the latter case, the results presented display the *n*-fold change in target gene mRNA-levels compared to their expression in mid-gestation, which was used as the non-treated control. GraphPad 3.06 (GraphPad Software, Inc., San Diego, California, USA) was used for all tests. Data are presented as the mean ± standard deviation.

### Immunohistochemistry

Our standard immunohistochemistry (IHC) procedure was applied on formalin-fixed, paraffin-embedded sections following the protocol described previously [12,17,25]. The list of primary antibodies and isotype controls is presented in Table 2. Briefly: after deparaffinization in xylol, slides were rehydrated in a graded ethanol series and washed under running tap water for 5 min. Antigen retrieval was performed in 10 mM citrate buffer pH 6.0 at room temperature for 5 min followed by microwave irradiation in an oven run at 560 W for 15 min and for 20 min at room temperature. Endogenous peroxidase activity was quenched by incubating the sections in 0.3% hydrogen peroxide in methanol for 30 min. The nonspecific binding sites were blocked with either 10% horse serum or 10% normal goat serum, depending on the secondary antibody used in experiments. Thereafter, slides were overlaid with primary antibodies and incubated overnight at 4°C. Two negative controls were performed: (a) omitting the primary antibody, and (b) antiserum-specific isotype controls at the same dilution and protein concentration as the primary antibody. The incubation with secondary antibodies was for 30 min at room temperature using 1:100 dilution. Following biotinylated secondary antibodies were used: horse anti mouse IgG BA-2000, goat anti-guinea pig IgG BA-7000, goat anti rabbit IgG BA-1000 and horse anti goat IgG BA-9500, all from Vector Laboratories Inc., Burlingame, CA 94010, USA. Signals were enhanced with the streptavidin-avidin-peroxidase Vectastain ABC kit (Vector Laboratories), for 30 min at room temperature. Peroxidase activity was detected with the DAB substrate Kit according to the manufacturer's instructions (Dako North America, Inc., City, Country). Post-staining was done using hematoxylin, slides were mounted in Histokit (Assistant Osterode, Germany).

**Table 2:** List of primary antibodies and isotype controls used for immunohistochemistry

**Name/antigen**	**Clone**	**Company**	**Immunogen**	**Concentration**	**Species/type**
**COX2**	clone 33	BD Pharmingen Heidelberg Germany	anti-rat COX-2 IgG	1:100	mouse monoclonal
**PGT**	(G-17) Sc-103085	Santa Cruz Biotechnology CA, USA	IgG against human PGT	1:100	goat polyclonal
**PGFS/AKR1C3**	custom made canine-specific antibody (Gram et al., 2013 [17])	Eurogentec Seraing Belgium	IgG against canine-specific peptide sequence DTLFATHPDYPFNDED, C-terminal amino acids 309–324	1:750	guinea pig polyclonal
**PGES**	custom made canine-specific antibody (Kautz et al., 2014 [26])	Eurogentec Seraing Belgium	IgG against canine-specific peptide sequence RSDQDVDRCLRAHRND, C-terminal amino acids 61–76	1:300	guinea pig polyclonal
**HPGD**	custom made canine-specific antibody (Gram et al., 2013 [17])	Eurogentec Seraing Belgium	IgG against canine-specific peptide sequence HFQDYETTPFHAKTQ, C-terminal amino acids 252–266	1:750	guinea pig polyclonal
**EP2**	catalog no. 101770	Cayman Chemicals MI, USA	IgG against human EP2, C-terminal amino acids 335–358	1:200	rabbit polyclonal
**EP4**	catalog no. 101775	Cayman Chemicals MI, USA	IgG against human EP4, C-terminal amino acids 459–488	1:100	rabbit polyclonal
**Isotype control**	IgG	Vector Laboratories Inc., Burlingame, CA, USA	−	Same protein concentration as primary antibody	mouse
**Isotype control**	IgG	Vector Laboratories Inc., Burlingame, CA, USA	−	Same protein concentration as primary antibody	rabbit
**Isotype control**	IgG	Vector Laboratories Inc., Burlingame, CA, USA	−	Same protein concentration as primary antibody	goat
**Isotype control**	IgG	Vector Laboratories Inc., Burlingame, CA, USA	−	Same protein concentration as primary antibody	guinea pig

## Results

### Semi-quantitation of uterine gene expression from pre-implantation until mid-gestation

Expression profiles of *COX2* (*PTGS2*), *PGFS/AKR1C3*, *FP* (*PTGFR*), *PGES*, the PGE2 receptors *EP2* and *EP4* (*PTGER2* and *PTGER4*), *HPGD*, the prostaglandin transporter (*PGT*, *SLCO2A1*) and the progesterone receptor (*PGR*) were assessed in uterine interplacental sites using semi-quantitative Real Time (TaqMan) RT-PCR.

**Figure 1:**
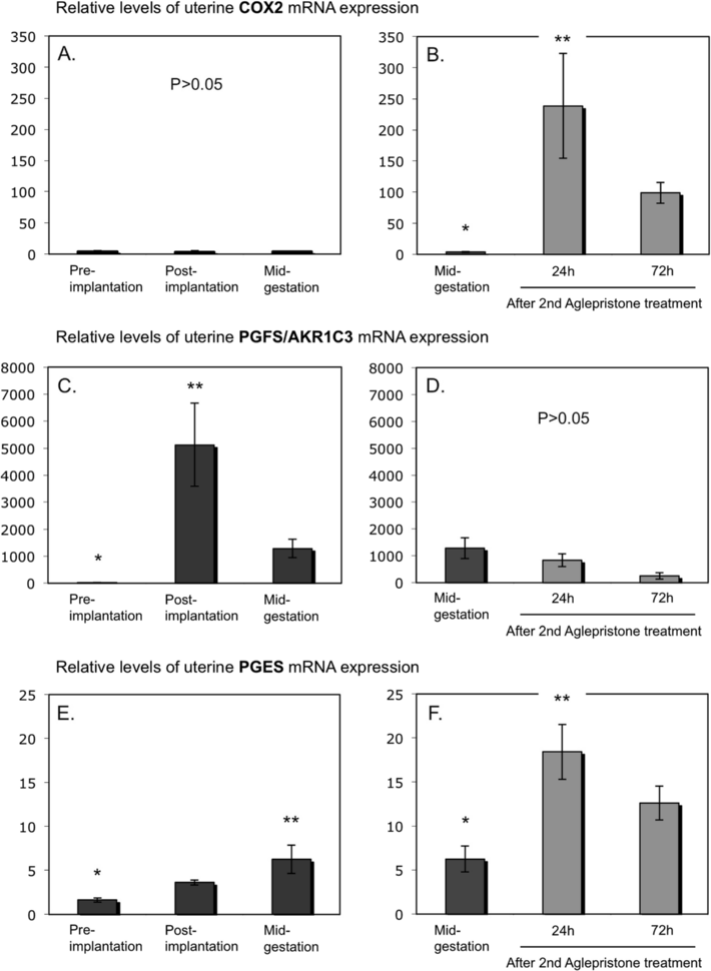
**Expression of cyclooxygenase 2 (COX2, PTGS2), prostaglandin F2α synthase (PGFS) and of prostaglandin E2 synthase (PGES).** Expression of COX2 (PTGS2), PGFS/AKR1C3 and PGES was determined by Real Time (TaqMan) PCR (mean ± SD) in the interplacental sites of canine uterus from the pre-implantation period until mid-gestation **(A,C,E)** and during Aglepristone®-induced luteolysis/abortion (**B,D,F**, compared with the mid-gestation group as a non-treated control). Bars with different asterisks differ either at P < 0.01 in **(C)** and **(E)**, or at P < 0.05 in **(B)** and **(F)**.

**Figure 2:**
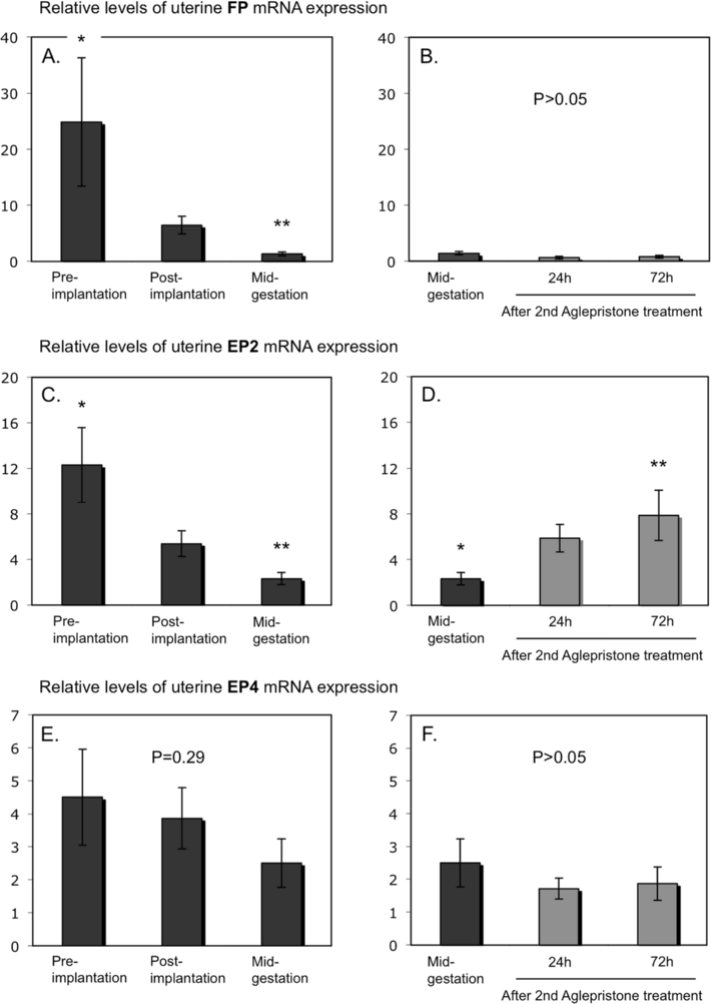
**Expression of PGF2α receptor (FP, PTGFR) and of PGE2 receptors, EP2 (PTGER2) and EP4 (PTGER4).** Expression of FP (PTGFR) and of EP2 and EP4 (PTGER2 and PTGER4, respectively) was determined by Real Time (TaqMan) PCR (mean ± SD) in the interplacental sites of canine uterus from the pre-implantation period until mid-gestation **(A,C,E)** and during Aglepristone®-induced luteolysis/abortion (**B,D,F**, compared with the mid-gestation group as non-treated control). Bars with different asterisks differ either at P < 0.01 in **(A)** or at P < 0.05 in **(C,D)**.

**Figure 3:**
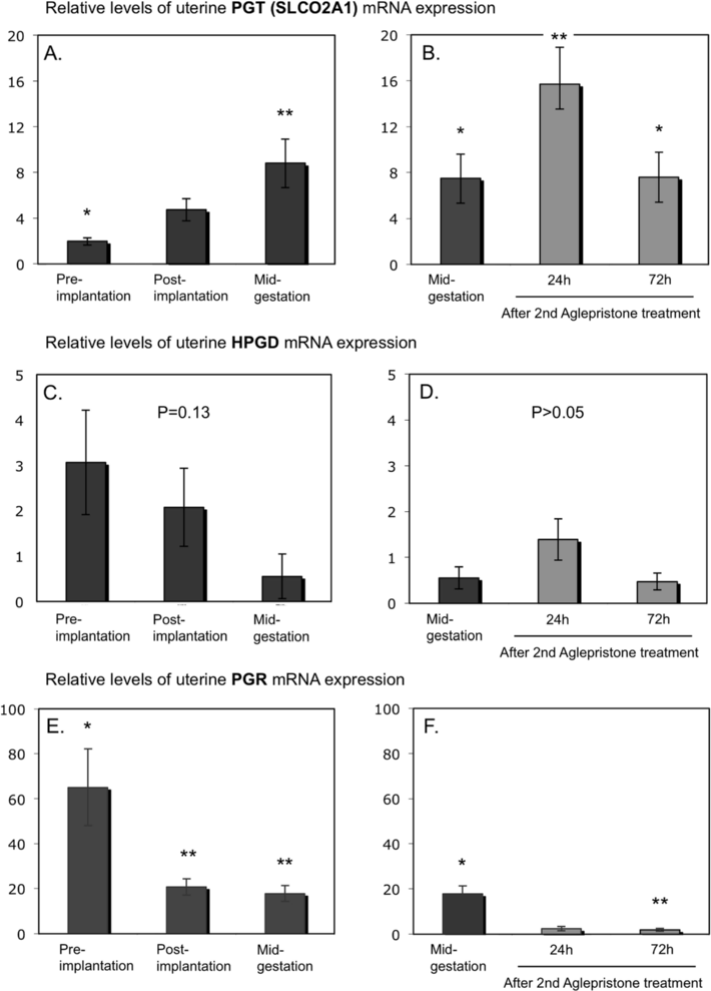
**Expression of prostaglandin transporter (PGT, SLCO2A1), 15-prostaglandin dehydrogenase (HPGD) and progesterone receptor (PGR).** Expression of PGT (SLCO2A1), HPGD and PGR was determined by Real Time (TaqMan) PCR (mean ± SD) in the interplacental sites of canine uterus from the pre-implantation period until mid-gestation **(A,C,E)** and during Aglepristone®-induced luteolysis/abortion (**B,D,F**, compared with the mid-gestation group as non-treated control). Bars with different asterisks differ either at P < 0.001 in **(F)** or at P < 0.05 **(A,B,E)**.

Whereas the expression of *COX2* was low and did not change from pre-implantation until mid-gestation (P = 0.92) (Figure 1A), the uterine expression of *PGFS/AKR1C3* was strongly modulated over time (P = 0.0005), increasing significantly from the pre-implantation to the post-implantation stage (P < 0.01) (Figure 1C), with the apparent decrease towards mid-gestation being not significant (P > 0.05). A significant effect of time (P < 0.0001) was observed for the *FP* receptor with values continuously decreasing from pre-implantation until mid-gestation (P < 0.01) (Figure 2A). There was a significant effect of time for *PGES* mRNA expression (P = 0.03) (Figure 1E), which increased gradually throughout the observational period, reaching the highest mRNA levels at mid-gestation (P < 0.01). Of the two PGE2 receptors, only the expression of *EP2* (*PTGER2*) changed significantly over time (P = 0.003), being significantly diminished at mid-gestation compared with pre-implantation (Figure 2C). In contrast, the expression of *EP4* varied widely among samples and remained statistically unaffected (P = 0.29) (Figure 2E). The expression of *PGT* (*SLCO2A1*) was significantly altered throughout the observational period (P = 0.01), increasing simultaneously with *PGES* mRNA and being higher in mid-pregnant dogs compared with the pre-implantation stage (P < 0.05) (Figure 3A). Although an apparent decrease was observed for *HPGD* mRNA expression with the progression of pregnancy, however, mostly because of very high variations in the specific mRNA content, the changes observed were statistically not significant (P = 0.13) (Figure 3C). As for *PGR* mRNA levels, after initially high expression during pre-implantation, they decreased afterwards, reaching significantly lower expression levels post-implantation and during mid-gestation (P < 0.05) (Figure 3E).

### The effects of antiprogestin treatment on uterine gene expression

Uterine gene expression was assessed by semi-quantitative Real Time PCR in uteri obtained from antigestagen-treated dogs. For all parameters, samples derived from the mid-gestation group served as non-treated controls in the statistical evaluation applying Dunnett’s multiple comparison test. A significant increase in *COX2* (*PTGS2*), *PGES* and *PGT* (*SLCO2A1*) expression was observed 24 hrs after the second treatment with Aglepristone® (P < 0.05) (Figure 1B, Figure 1F and Figure 3B). The increase in *EP2* (*PTGER2*) levels was more pronounced (P < 0.05) after 72 h (Figure 2D). An apparent suppression of PGR expression was already observed 24 h after the antigestagen treatment and it was significantly downregulated (P < 0.001) at 72 h (Figure 3F). The expression of *PGFS/AKR1C3*, *FP* (*PTGFR*), *EP4* (*PTGER4*) and *HPGD* was unaffected (P > 0.05) (Figure 1D, Figure 2B, Figure 2F, Figure 3D).

### Immunohistochemical (IHC) localization of gene expression

**Figure 4:**
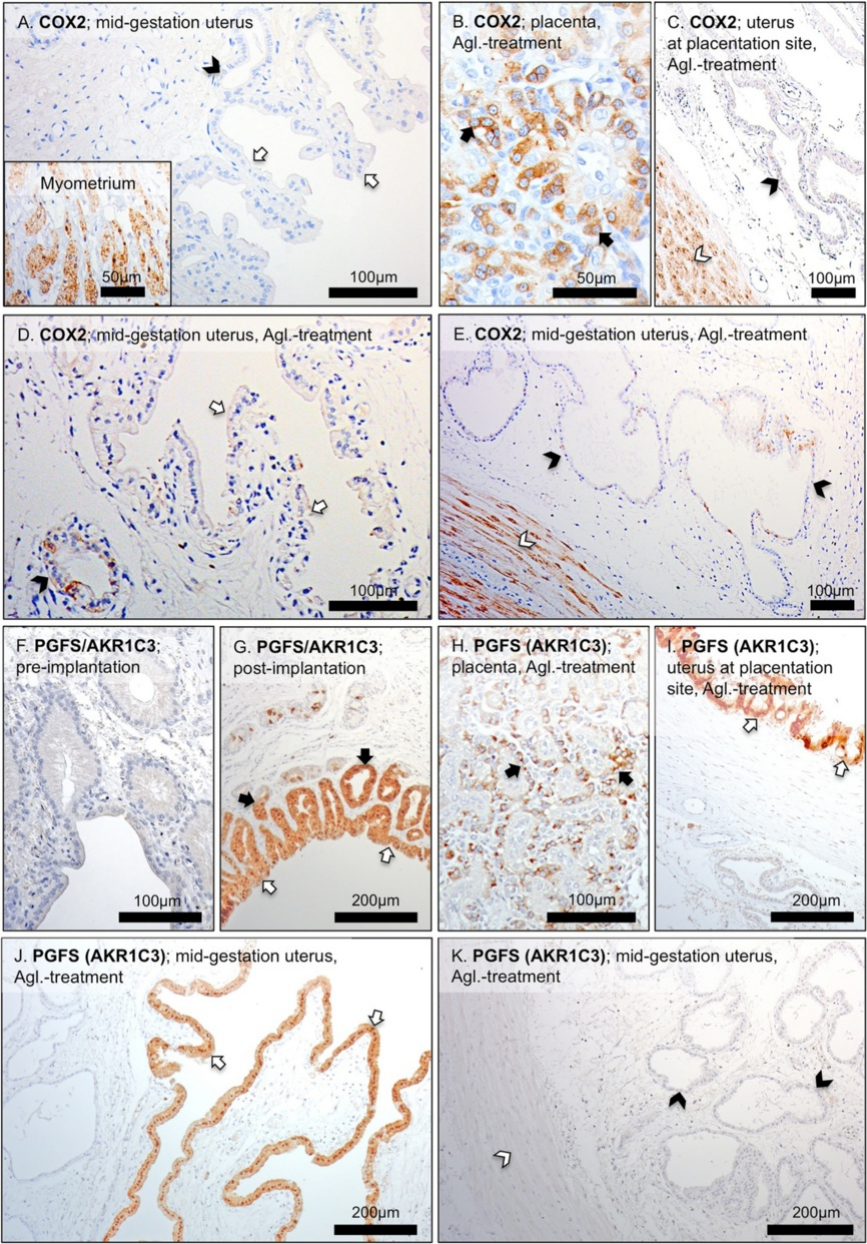
**Immunohistochemical localization of cyclooxygenase 2 (COX2, PTGS2) and prostaglandin F2α synthase (PGFS/AKR1C3).** Immunohistochemical (IHC) localization of COX2 (PTGS2) at interplacental uterine sites during mid-gestation **(A)**, as well as in the utero-placental compartment **(B,C),** and interplacental uterine sites **(D,E)** during Aglepristone® (Agl.)-induced abortion. IHC localization of PGFS/AKR1C3 in the uterus pre-implantation **(F)**, post-implantation **(G)** and in the utero-placental compartment **(H,I)** and interplacental uterine sites **(J,K)** during Aglepristone® (Agl.)-induced abortion. Whereas no or only very weak COX2 IHC signals are observed in uterine endometrium (open arrows = surface luminal epithelium, solid arrowhead = uterine gland), clearly visible staining is localized in myometrium **(A)**. In the utero-placental compartment during Agl.-induced parturition, placental COX2 is localized to the fetal trophoblast cells (solid arrows in **B**); there are only very weak signals in the uterine glands (solid arrowhead in **C**), but strong ones are present in myometrium (open arrowhead in **C**). In the interplacental sites, following Agl. treatment, strong COX2 staining is localized in myometrium (open arrowhead in **E**), and only very weak staining is present in surface (luminal) uterine epithelium (open arrows in **D**) and uterine glands (solid arrowheads in **D** and **E**). No or only very weak uterine signals are observed for PGFS/AKR1C3 pre-implantation **(F)**. Following implantation, PGFS/AKR1C3 protein is localized in surface (luminal) uterine epithelium and endometrial superficial glands (open and solid arrows in **G**). In the utero-placental compartment during Agl.-induced parturition, placental PGFS/AKR1C3 protein is localized to the fetal trophoblast cells (solid arrows in **H**); strong signals are localized also in the superficial endometrial glands, the so-called glandular chambers (open arrows in **I**). Within the interplacental uterine sites, in Agl.-treated animals, PGFS/AKR1C3 staining is localized predominantly in surface (luminal) uterine epithelium (open arrows in **J**); the open and solid arrowheads in **(K)** indicate myometrium and uterine glands, respectively, with barely detectable signals.

**Figure 5:**
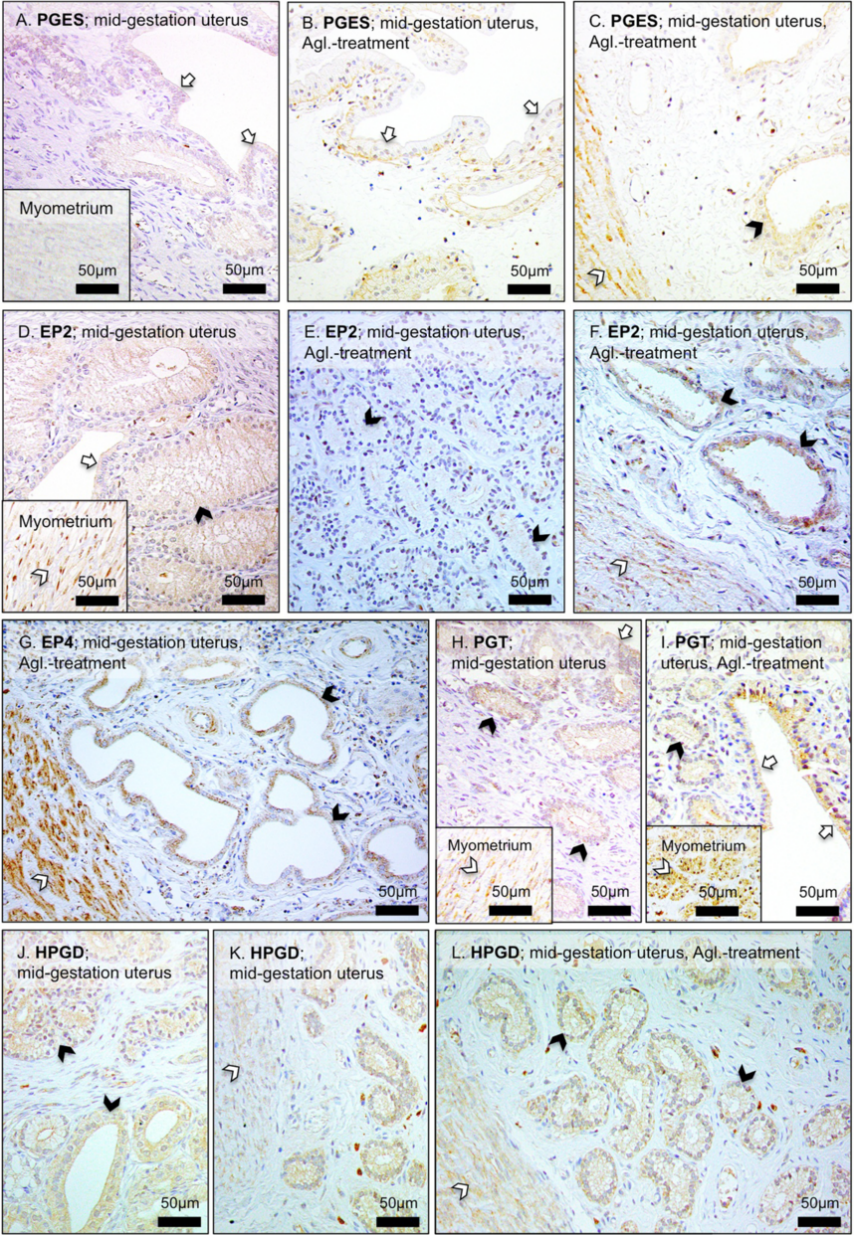
**Immunohistochemical localization of prostaglandin E2 synthase (PGES), PGE2 receptors EP2 and EP4 (PTGER2 and PTGER4), prostaglandin transporter (PGT, SLCO2A1) and of 15-prostaglandin dehydrogenase (HPGD).** Immunohistochemical (IHC) localization of: **(A-C)** PGES, and **(D-F)** EP2 (PTGER2) in interplacental uterine sites during mid-gestation and during Aglepristone® (Agl.)-induced abortion. **(G)** Representative picture of EP4 (PTGER4) expression in interplacental site during Aglepristone® (Agl.)-induced abortion in mid-gestation dog. **(H,I)** Expression of PGT (SLCO2A1) in interplacental uterine site during mid-gestation and during Aglepristone® (Agl.)-induced abortion. **(J-L)** Uterine expression of HPGD in interplacental uterine sites during mid-gestation and after Aglepristone® (Agl.) treatment. Open arrows = surface (luminal) uterine epithelium, solid arrowheads = uterine glands, open arrowheads in **(C)**, **(F)**, **(K)**, **(L)** = myometrium.

All antibodies applied in this study were used in our previous projects and found to be specific for the canine species [15,17,26]. No or only very weak IHC signals were observed in the uterine endometrium for COX2 (PTGS2), whereas myometrium stained strongly in all samples investigated (Figure 4A). A similar signal distribution pattern was observed following the antigestagen treatment (Figure 4D,E). Additionally, utero-placental cross-sections (full thickness, middle part of the placental girdle avoiding marginal hematoma) obtained from the same animals in which abortion was induced with an antigestagen were included in the experiment and stained, revealing the COX2 (PTGS2) protein expression pattern previously described [15], i.e., strong COX2 signals were spread over the entire trophoblast during induced parturition coinciding with a substantial prepartum increase of PGF2α, while only weak signals were visible in the adjacent endometrial tissues (Figure 4B,C). After low and only barely detectable expression of PGFS/AKR1C3 protein during the pre-implantation stage of pregnancy, its uterine expression was clearly detectable, displaying a tendency towards over-staining, post-implantation and was localized predominantly to the superficial luminal endometrial epithelium (Figure 4F,G), while only weak staining was observed in the uterine glands and in myometrium (not shown); distribution pattern and staining intensity did not change in Aglepristone®-treated dogs (Figure 4J,K). As for COX2 (PTGS2), the expression pattern of PGFS/AKR1C3 protein in the interplacental sites was comparable with its previously published [17] distribution in the utero-placental compartment, revealing the co-localization of PGFS/AKR1C3 with COX2 expression (Figure 4H,I).The expression of PGES (Figure 5A-C) showed a similar distribution pattern from the pre-implantation until mid-gestation and at induced parturition-derived uterine samples, with signals localized in endometrial epithelial cells, weaker staining in the uterine stroma, and myometrial signals, which became clearly detectable at induced parturition (Figure 5C). A similar staining pattern was observed for the two PGE2-receptors, EP2 and EP4, with signals evenly distributed between the endometrial and myometrial compartments (Figure 5D-G). The expression of PGT (SLCO2A1) was co-localized with other PG-system members, however, with myometrial signals, appearing stronger following the antigestagen treatment (Figure 5H,I). A similar localization pattern, but with widely varying IHC signals among individual animals, was noted for HPGD (Figure 5J). As for EP2 and EP4, the signal distribution and intensity did not change during induced preterm parturition/abortion (Figure 5K,L).

## Discussion

By showing strong functional interrelationships, and to some extent opposite effects, prostaglandins (PGs) unequivocally play an important role in regulating reproductive functions. Thus, whereas PGE2 was shown to be involved, e.g., in softening the cervix during parturition [27,28], PGF2α is the best-known luteolytic factor, while both PGs are involved in coordinating myometrial contractile activity [29-31]. Accordingly, uterine expression of the respective genes encoding for factors involved in PGs synthesis and metabolism, the so-called prostaglandin system, was shown in several species, such as cattle [22], sheep [32,33] and rats [34].

In the present study, the expression and localization of the PG-system was investigated in canine interplacental uterine tissues at selected time points of pregnancy, i.e., pre-implantation, post-implantation and mid-gestation (there was no tissue material available for experiments from the interplacental sites from dogs during normal perpartum luteolysis).

Our data clearly indicate the basic capability of these tissues to synthesize and respond to prostaglandins, because the expression of all major components of the PG-system was detectable in all tissue samples investigated, both at the mRNA and protein level.

In general, the gene expression patterns observed resembled their expression in the corresponding utero-placental compartments [15,17]. The expression of *COX2* remained low until mid gestation, and the post-implantation period was characterized by increased *PGFS/AKR1C3* expression. This, together with the gradually rising *PGES* expression levels and the concomitant presence of the respective prostaglandin receptors, imply a local role of these hormones during canine decidualization and implantation, as indicated previously [15,17]. Also, in agreement with our previous conclusion [17], a locally increased *HPGD* expression during earlier stages of pregnancy, which - in the present study - shows up as a tendency, could additionally restrict and coordinate the endocrine/paracrine effects of PGs.

The antigestagen-mediated blocking of the uterine PGR receptor, whose expression was significantly downregulated after implantation, resulted in upregulated uterine *COX2* and *PGES* expression, thereby resembling their expression patterns in utero-placental compartments during normal and preterm parturition/abortion [15]. Interestingly, however, their localization patterns did not change significantly in response to the antigestagen treatment and, especially for COX2, the strongest signals were still observed in the myometrium. This was compared with their distribution pattern in the corresponding utero-placental compartments, confirming their presence in the fetal part of the placenta, i.e., in the trophoblast cells, as described in our previous research [15,17]. Less dramatic changes, but also with an overall expression pattern resembling that observed in utero-placental samples, pertain to the uterine expression of *PGFS/AKR1C3*, *FP*, *EP4* and *HPGD* in antigestagen-treated bitches.

Consequently, based on our observations, we infer that the upregulated uterine expression of COX2 and PGES may originate mostly in the myometrial compartment, because their localization pattern did not differ significantly following treatment with Aglepristone®. This is in contrast to observations made in cattle where the strongly upregulated COX2 expression was predominantly localized in surface epithelial cells, both in animals undergoing spontaneous labour and those in which it was induced prematurely [22,35]. However, as in cattle, in dogs PGs derived from the interplacental uterine sites seem to contribute primarily to myometrial contractility in conjunction with fetal expulsion, with the utero-placental compartment, however, remaining an important source of PGs around term [36,37].

The alterations in placental feto-maternal communication occurring in response to the local withdrawal of progesterone appear to be important for subsequently increasing output of the luteolytic hormone PGF2α, which with respect to the placenta must originate in the fetal component, i.e., in the trophoblast cells, where PGs synthesizing enzymes are expressed and localized. This conclusion seems to relate to both the bovine and canine species [15,36,37].

Furthermore, also in accordance with our previous studies [15], the upregulated expression of COX2 during induced parturition points towards the substantial role of COX2 (PTGS2) as a rate-limiting factor in provision of prepartum prostaglandins in the dog. Such a role of COX2 as a rate-limiting factor has also been observed in the horse [38], where blocking of endometrial COX2 expression by the conceptus at day 15 of early pregnancy prevented PGF2α-induced luteal regression and resulted in continuation of pregnancy.

At the functional level, our descriptive findings concerning the basal capability of canine uterine and placental tissues to produce and respond to PGs, are not conclusive and will require further studies including, e.g., measurements of the local PGs content within those tissues during different stages of canine gestation.

## Conclusions

Our study presents the expression and localization patterns of factors involved in the synthesis of prostaglandins, the so-called prostaglandin-system, in canine interplacental uterine sites at selected times of pregnancy (pre-implantation, post-implantation and mid-gestation). Additionally, changes in the expression and availability of these factors and, hence, their potential contribution to the process of parturition, have been investigated in dogs in which progesterone receptor function was blocked by an antigestagen.

Based on the observations presented herein, in addition to prostaglandins of placental origin, the canine pregnant uterus also appears to be an organ actively involved in prostaglandin synthesis during canine parturition, most probably contributing predominantly to myometrial contractility. From a practical point of view, our findings can help us to better understand the mechanisms responsible for parturition in the dog, especially because decreased synthesis of prostaglandins and/or reduced uterine sensitivity to them could result in serious clinical conditions such as uterine inertia.

## Competing interests

The authors declare that they have no competing interests.

## Authors’ contributions

MPK: Idea of the study, collecting tissue samples, Real Time PCR experiments, statistical evaluation, interpretation of data, manuscript writing. EK: RNA isolation, Real Time PCR experiments, immunohistochemical procedures. EH: Tissue preparation and processing, immunohistochemical procedures. BH: coordination of animal experiments and collection of tissue samples, knowledge transfer, critical discussion of the data, editing of the manuscript. AB: knowledge transfer, critical discussion of the data, editing of the manuscript. All authors read and approved the final manuscript.
